# The phenomenology of auditory verbal hallucinations in bipolar disorder

**DOI:** 10.1111/papt.12446

**Published:** 2023-02-07

**Authors:** Lindsay M Smith, Caitlin Yolland, Susan L. Rossell, Wei Lin Toh

**Affiliations:** ^1^ National and Specialist CAMHS, At‐Risk and Forensic Service, South London and Maudsley NHS Foundation Trust, Michael Rutter Centre Maudsley Hospital London UK; ^2^ Department of Psychology, Institute of Psychiatry, Psychology and Neuroscience King's College London London UK; ^3^ Centre for Mental Health Swinburne University of Technology Melbourne Victoria Australia; ^4^ Department of Psychiatry St Vincent's Hospital Melbourne Victoria Australia; ^5^ Department of Psychology Alfred Hospital Melbourne Victoria Australia

**Keywords:** auditory hallucinations, bipolar disorder, depression, hearing voices, mania

## Abstract

**Objectives:**

At least one in four persons with bipolar disorder (BD) are estimated to have experienced auditory verbal hallucinations (AVH) or heard voices at some point. Yet few studies have investigated AVH in detail in this population. This preliminary study examined the phenomenology of AVH in BD to identify commonalities and differences relative to other psychiatric disorders where AVH are commonly reported.

**Method:**

Twenty‐one participants diagnosed with BD were recruited across two international sites in the UK and Australia. All participants underwent a structured clinical interview to verify psychiatric diagnosis and completed standardised measures of symptomatology, including mood states. Phenomenological information of AVH was gathered using select questions from the comprehensive Mental Health Research Institute Unusual Perceptual Schedule (MUPS).

**Results:**

AVH experienced by this BD sample were broadly similar in form and content to characterisations reported in the schizophrenia spectrum disorders (SSD) in prior literature, with some exceptions including frequency, duration and the changeability of tone and content.

**Conclusions:**

The study highlights possibly subtle differences in the experience of AVH in BD, including the potential influence of mood congruence as a pertinent clinical feature. Further research into these differences might inform adaptations to existing AVH interventions to ensure they are relevant for BD.

**Practitioner points**

- The study reports a detailed phenomenology of Auditory Verbal Hallucinations (AVH) in Bipolar Disorder (BD) using the Mental Health Research Institute Unusual Perception Schedule.
- AVH experiences in BD are broadly similar to those observed in SSD, with some exceptions in the frequency, duration and the changeability of tone and content.
- There is an important influence of mood congruence in AVH experiences in BD.
- Assessment over time of AVH in BD is needed to capture fully the dynamics between mood and psychotic symptoms.

## INTRODUCTION

Auditory verbal hallucinations (AVH) or ‘hearing voices’ have been defined clinically as the perception of verbal utterances in the absence of corresponding external stimuli (Slade & Bendall, 1988). These represent complex experiences that may be described across diverse phenomenological facets, including perceptual (e.g. frequency, duration, sound quality), cognitive (e.g. conviction, interaction), and emotional (e.g. valence, distress) characteristics. AVH complexity is such that experiential reports also include those of ‘soundless voices’ or a sense of being communicated to without any ‘real’ sound quality (Humpston & Broome,  2015). Our conceptual understanding of AVH facets is therefore debatable and constantly evolving. The current study aimed to describe and explore certain phenomenological parameters of AVH in bipolar disorder (BD), where this area of research has been comparatively overlooked.

### Current conceptualisations of AVH experiences: negative or not?

Experiences of AVH need not always be a cause for clinical concern nor precipitate help‐seeking behaviours (McCarthy‐Jones, 2012). Throughout history, ‘voice hearing’ experiences have been deliberately induced with the aid of psychotropic substances, including by Beat generation poets invoking inspiration and by Siberian Shamans seeking spiritual guidance (Laroi et al., 2014). A certain proportion of the general population, estimated at 5%–15%, report AVH in a form that is typically held as benign (Beavan et al., 2011; Toh et al., 2022). For those with mental health conditions, voice‐hearing can also at times be pleasurable (Sanjuan et al., 2004). However, negative AVH experiences remain the most common hallucinatory phenomenon in psychotic disorders and are often associated with significant emotional distress, functional disturbance and clinical risk (Thomas et al., 2014). Up to 80% of individuals with a schizophrenia spectrum disorder (SSD) report hearing lifetime voices, alongside a significant proportion of those with other mental health conditions such as BD or borderline personality disorder (Laroi et al., 2012; Merrett et al., 2016; Toh et al., 2022). Owing to their prominence and diagnostic significance in SSD, AVH have been well‐researched in that clinical cohort, but less is known about the experience and impact in other psychiatric groups, including BD (Smith et al., 2017).

### Phenomenology of AVH in BD: what we know:

Bipolar disorder is characterised by periods of elated mood (i.e. mania or hypomania) and depression that can be accompanied by psychotic symptoms (Goodwin & Jamison, 2007). As such acute mood episodes in BD are diagnosed within key international disease classification systems as ‘with or without psychotic features’, based on co‐occurring hallucinations or delusions (American Psychiatric Association, 2013; World Health Organisation, 2018). Rates of AVH in BD have ranged widely from 11% to 67% (Laroi et al., 2012; Pini et al., 2004), with empirical evidence corroborating that voice‐hearing during the extreme mood states that form a core element of the disorder tends to be more common (Braunig et al., 2009; Nisha et al., 2015). Furthermore, there is developing consensus that AVH in BD seem to be associated with manic or mixed mood episodes more often than in depression (Azorin et al., 2013; Baethge et al., 2005). Despite this apparent frequency and co‐occurrence with acute illness episodes, few studies have attempted to investigate specific characteristics of AVH in BD, for instance, delving into the form and content of these voices as well as their emotional impact and encompassing unique aspects such as mood congruence (Laroi et al., 2012; Toh et al., 2015).

A small number of exceptions exist. A study by Kumari et al. (2013) compared dimensions of AVH in 30 persons with affective psychosis (22 of whom had BD) with a SSD sample. Those with affective psychosis scored less poorly on a range of phenomenological facets encompassing frequency, duration and negative content, though notably they reported significantly greater distress intensity. Similarly, Okulate and Jones (2003) found that their affective psychosis sample (*n* = 13; number with BD unknown) were more likely to experience voices that invoked fear and were more likely to be complied with relative to a SSD group (*n* = 76). Both studies were limited by small or mixed samples, as is common, and which limited the specificity of results to BD. Added issues, including cross‐cultural generalisability and the reliability of some measures, indicate a need for further research into AVH phenomenology in BD to replicate and explain these nascent findings. Only with more nuanced characterisations of AVH subtypes across disorders will we be able to spur development in aetiological models explaining their pathogenesis, and subsequently, foster more tailored and effective treatments (McCarthy‐Jones et al., 2014).

### Aims and hypotheses of the current study

A primary aim of the current study was to provide detailed descriptive phenomenology of AVH in BD, given scant existing literature. The key research question was: What are the core phenomenological features of AVH in BD, relative to what is known about these experiences in SSD? Elements of experience that directly pertain to major clinical features of BD, for example involving mood congruence, were also of special interest.

## METHOD

### Participants and procedure

Participant eligibility criteria were: (i) aged 18 years or above, (ii) with a primary BD diagnosis (see Psychiatric diagnoses and comorbidities section), (iii) sufficient English language ability to complete the protocol, (iv) adequate recall of AVH experiences to provide meaningful detail, (v) AVH frequency of at least once a day over three distinct episodes, and (vi) no intellectual disability or significant neurological disorders. To maintain ecological validity, comorbid psychiatric disorders and/or history of substance use disorders, if remitted >12 months prior, were permitted (nb. if AVH occurred exclusively during periods of substance use, participants were excluded). Distinct streams of recruitment were employed across the two sites. At the UK site, this comprised: (i) the National Institute for Health and Care Research, Maudsley Biomedical Research Centre's Consent for Contact initiative operating within South London and the Maudsley National Health Service (NHS) Foundation Trust, (ii) outpatient recovery services within the same NHS Trust, and (iii) Bipolar UK non‐profit organisation. At the Australian site, this comprised: (iv) public and private mental health services in Victoria, (v) the Voices Research Participant Registry held at Swinburne University of Technology, and (vi) community advertising via local venues or online research participation forums.

In total, 21 participants with a primary diagnosis of BD were recruited. All were stabilised in the community at various stages of recovery, except for one, who attended in an acutely manic state (nb. this person was admitted as an inpatient, where they opted to continue the study). Participants completed a standardised in‐person clinical interview as well as self‐report questionnaires on AVH (nb. an option involving telephone interview, alongside postal questionnaires was possible, if preferred). Each session took less than three hours, and all participants provided informed consent. Ethics approvals were respectively received from the UK National Research Ethics Committee (London and City East, 15/LO/0205) and Swinburne University of Technology Research Ethics Committee (R/2018/215).

### Measures

Basic demographic (e.g. age, sex) and health (e.g. illness history, medication use) information was collected. The following measures were respectively employed to ascertain psychiatric diagnoses and comorbidities, mood symptoms as well as AVH phenomenology.

#### Psychiatric diagnoses and comorbidities

The Mini International Neuropsychiatric Interview (MINI; Sheehan et al., 1998) is a structured diagnostic interview, allowing for a brief but accurate screen for 20 major psychiatric diagnoses, with a <30 min administration time, depending on number of subsections employed (Modules A‐N for the current study; nb. psychiatric diagnoses were further verified using electronic patient records, where permissible).

#### Mood symptoms

Owing to site operational differences, two discrete (but comparable) sets of mood measures were employed. At the UK site, the Altman Self‐Rating Mania Scale (ASRMS; Altman et al., 1997) and Quick Inventory of Depressive Symptomatology Self‐Rated (QIDS‐SR; Rush et al., 2003) were used. The ASRMS assesses the severity of manic or hypomanic symptoms across five domains (i.e. *happiness*, *self‐confidence*, *sleep*, *speech* and *activity level*), rated on five‐point Likert scales (0–4). Scores are summed, with higher scores denoting increased mania severity (0–20), and a cut‐off ≥6 indicating probable mania or hypomania. The QIDS‐SR gauges depressive symptoms across nine domains (i.e. *sad mood*, *concentration*, *self‐criticism*, *suicidal ideation*, *interest*, *energy/fatigue*, *sleep disturbance*, *appetite/weight changes*, and *psychomotor agitation/retardation*), rated on four‐point Likert scales (0–3). The largest score in each domain is summed, with higher scores denoting increased depressive severity (0–27). Categorical ratings denote: ≤5 *no depression*; 6–10 *mild depression*; 11–15 *moderate depression*; 16–20 *severe depression*; and ≥21 *very severe depression*.

At the Australian site, the Young Mania Rating Scale (YMRS; Young et al., 1978) and Beck Depression Inventory (BDI‐II; Beck et al., 1961) were used. The YMRS assesses the severity of manic or hypomanic symptoms across 11 domains (i.e. *elevated mood*, *increased motor activity‐energy*, *sexual interest*, *sleep*, *irritability*, *speech*, *language‐thought disorder*, *content*, *disruptive‐aggressive behaviour*, *appearance* and *insight*), rated on five‐point Likert scales (0–4). Scores are summed (with four items double‐weighted), with higher scores denoting increased mania severity (0–60). Categorical ratings denote: ≤12 *remission*; 13–19 *minimal symptoms*; 20–25 *mild mania*; 26–37 *moderate mania*; and ≥38 *severe mania*. The BDI‐II is a 21‐item self‐report measure of depressive symptoms, assessing emotional, cognitive, motivational and physiological domains of depression. For each scale, higher scores denoted increased symptomatology and validated categorical clinical threshold ratings were available., rated on four‐point Likert scales (0–3). Scores are summed, with higher scores denoting increased depressive severity (0–63). Categorical ratings denote: ≤9 *no depression*; 10–18 *mild depression*; 19–29 *moderate depression*; and ≥30 *severe depression*. Good convergent validity has been documented between the ASRMS and YMRS (Altman et al., 1997) as well as the QIDS‐SR and BDI‐II (Rush et al., 2003).

#### Phenomenology of AVH

The Mental Health Research Institute Unusual Perception Schedule (MUPS; Carter et al., 1995) is the most comprehensive semi‐structured interview assessing individual AVH experiences to date. It comprises 365 questions recording descriptive features across seven major domains: *physical characteristics* (e.g. tone, volume), *personal characteristics* (e.g. gender, identity), *relationship to voices and feelings* (e.g. emotional impact), *form and content*, *cognitive processes* (e.g. relationship to delusions), *perceptions/personal views* (e.g. reality), and *psychosocial issues* (e.g. coping). There is also a subsection on *mood congruence* of particular relevance to the current study. Depending on the specific question, response formats can be open‐ended or fixed (e.g. categorical, Likert‐style). A select subset of MUPS items, coinciding with the most common AVH phenomenological facets reported in SSD, were analysed. To ensure consistency, participants were asked to recount their most salient or memorable AVH experience.

### Statistical analyses

Data analysis was performed with IBM SPSS Statistics, v.27. Data were presented as means and standard deviations or percentages (whichever was most meaningful). Preliminary analysis of demographic and clinical information helped to characterise our sample. To answer our primary research question examining the phenomenology of AVH in BD, descriptive statistics were reported.

## RESULTS

Participant demographic and clinical information is shown in Table 1. Mean age of participants was in their 40s, with a slight overrepresentation of males, largely born in an English‐speaking country, with most not engaged in paid employment and unpartnered. The majority were taking mood‐stabilising medication, with slightly over a third ascribing to probable mania or hypomania symptoms at time of assessment, and participants displaying a range of depression severities.

**TABLE 1:** Participant demographic and clinical information (*N* = 21).

	Mean ± standard deviation or proportion in % (*n*)
Demographic	
Mean age (years)	43.5 ± 12.8
Sex (% male)	57.1 (12)
Birth country (% English‐speaking)	90.5 (19)
Employment (%)	
Full‐time paid	23.8 (5)
Part‐time paid	9.5 (2)
Unemployed/sick leave/incapacity benefit	38.1 (8)
Retired/volunteering/studying	28.6 (6)
Marital (%)	
Single	61.9 (13)
Married/de facto partnership	28.6 (6)
Divorced/separated	9.5 (2)
Clinical	
Number of inpatient admissions	3.9 ± 2.6
Mean illness duration (weeks since first admission)	825.0 ± 515.9
Mood‐stabilising medication (% yes)	95.2 (20)
Mania or hypomania symptoms	AMSRS = 5.6 ± 4.8 (*n* = 17); YMRS = 6.4 ± 3.8 (*n* = 5)
Probable (%)	38.1 (8)
Depressive symptoms	QIDS‐SR = 12.7 ± 7.2 (*n* = 17); BDI‐II = 18.2 ± 16.0 (*n* = 5)
None (%)	19.0 (4)
Mild (%)	33.3 (7)
Moderate (%)	14.3 (3)
Severe (%)	19.0 (4)
Very severe (%; only for QIDS‐SR)	14.3 (3)

Abbreviations: AMSRS, Altman Mania Self‐Rating Scale; QIDS‐SR, Quick Inventory of Depressive Symptomatology Self‐Rate; YMRS, Young Mania Rating Scale.

### Physical characteristics of AVH

A comprehensive table detailing the proportions of participants endorsing specific phenomenological facets of AVH based on select MUPS items can be found in Table S1 (see Supporting Information); only pertinent findings are described here. First age of AVH onset in the BD sample was most commonly between 20 and 29 years (38.1%); almost a third (28.6%) were under 14 years old; and nearly a fifth (19.0%) were over 30 years old [Item 2]. More than half of participants (52.5%) had heard voices within the past 12 months [Item 3]. During their most salient or memorable AVH experience, most participants (66.6%) reported voices as *often* or *constantly with you* (sic); and a third experienced voices *rarely* or *occasionally* [Item 4]. Participants were equally split three‐ways in terms of those who experienced their voices as *internal*, *external* or *both internal and external* [Item 8]. Dominant voices were commonly experienced at *normal* conversational volumes (57.1%), though at their loudest, were often *loud* or even *yelling/screaming* (57.1%) [Item 11]. Voices were almost always rated as *clear* or *very sharp/unusually clear* (90.5%), though 38.1% of the sample stated that the content was *incomplete* or *indistinguishable* at times [Item 13].

### Form and content of AVH

The most common form of address was second‐ or implied second‐person (71.4%) [Item 16]. In addition, significant proportions of participants also *sometimes* or *often* heard voices conversing about them (42.8%) [Item 19] or giving a running commentary of their thoughts or actions (47.6%) [Item 21]. Slightly over half of participants (52.4%) experienced voices that *sometimes* or *often* told them what to do, with more than a third (38.1%) *sometimes* or *often* not able to resist such commands [Item 25].

Participants were asked to reflect on the tone and content of their most dominant voice, and to select as many appropriate descriptors as applicable, based on the list of adjectives provided [Items 12 and 22]. Most participants endorsed multiple tones, which were largely negative, using descriptors such as *harsh* (47.6%), *malicious/nasty* (42.6%), *angry* (33.3%) or *menacing* (33.3%). Somewhat neutral words were also selected, including *authoritative* (52.4%), *sharp* (52.4%) or *bossy* (33.3%), whereas a small proportion reported positive tones, which were *gentle* (28.6%), *kind* (28.6%), *friendly* (28.6%) or *loving* (14.3%). Similarly, the majority described their voice content mostly in negative terms, including *critical* (57.1%), *intrusive* (52.4%) or *derogatory* (47.6%), with a small fraction endorsing positive voice content, such as *affirming* (23.8%), *guiding* (19.0%) or *inspiring* (14.3%). Yet even those who selected positive descriptors for voice content clarified upon further reflection that these experiences may not be positive per se, as their voices could be “cajoling” or “conspiring” with undesirable actions [Participants 21 and 12]. Moreover, the valence of voice tone and content were not always congruent, with 23.8%–38.1% of the sample reporting that the tone or content of their voices had changed over time.

### Perception/personal views of AVH

The majority of participants (85.7%) rated the reality of their voices as *very real* [Item 27]. A third acknowledged that the mood of their voices was like their own mood at the time at least *sometimes* or *often* [Item 37]. For the others however, the opposite was true, where for instance a participant shared that: “she was in a different mood than me at the time, she was more stronger (sic) than I was at that time, was able to comfort me” [Participant 6].

## DISCUSSION

The current study aimed to examine the phenomenological features of AVH in BD, and compare these with commonly reported AVH characteristics in SSD. With respect to our primary research question, phenomenological information derived from the MUPS indicated that AVH within BD may be broadly similar in form and content to typical experiences characterised within SSD (McCarthy‐Jones & Resnick, 2014; Toh et al., 2020). Certainly, the results testify to the diversity and complexity of the AVH phenomenon (McCarthy‐Jones, 2015). These findings also bear notable clinical implications, where several observations raise questions for ongoing research. First, AVH were reported within this BD sample as occurring both within and outside of acute mood episodes. Whilst the typical experience was of a constant voice during a past mood episode, approximately a third of the sample described current occasional voices. This observation is perhaps relevant to the ongoing consideration of how best to conceptualise the relationship between BD and other diagnostic conditions where AVH are experienced alongside mood and emotional difficulties, including SSD and borderline personality disorder. Although historically considered to be separate disease entities, there has been much written about the implications of studies showing common phenomenological and neurobiological overlap in BD and SSD of late (Craddock & Owen, 2010; Pearlson, 2015). Keshavan et al. (2011) have devised a brief descriptive scale, based on type and relative proportion of symptoms over the illness course, to capture interactions between psychosis and affective symptoms dimensionally. Current findings would support the development of such continuum or symptom‐based approaches to understanding AVH across BD and SSD, alongside independent disease‐based models, without necessarily undermining the practical and clinical value of categorical diagnoses.

Second, voices with positive, neutral and negative tone and content, not necessarily congruent in terms of valence, were described in the current sample. Only a third of participants acknowledged that the mood of their voices was similar to their own mood at the time. Incongruence in positivity or negativity ratings of AVH tone versus content (and mood) suggest the need for revision of the criteria for mood congruence. Shortcomings of this construct have also been noted elsewhere (Toh et al., 2015). At present, the definition of mood congruent psychotic symptoms in BD refer only to voice content and evaluate mood congruence on objective observation (American Psychiatric Association, 2013; World Health Organisation, 2018). Mood congruence criteria may thus be better operationalised by referring to specific elements of AVH phenomenology, including how voices are experienced by a person relative to their mood, rather than clinical judgement alone.

Third, voices conversing or providing a running commentary were relatively common in this sample, consistent with research that has led to abandonment of Schneiderian ‘first rank’ symptoms as specific to SSD (Upthegrove et al., 2016). A majority of the current BD sample endorsed voices comprising second‐person address, as also previously documented (Kumari et al., 2013). That voices in BD may be more likely to address one directly using ‘you’ has definite clinical implications. Direct statements to an individual may influence how they conceive of their relationship with voices, and concomitant levels of engagement and compliance. Indeed, AVH addressing people in the second person were found to be significantly more unpleasant than those that did not (Copolov et al., 2004).

### Limitations of the current study

Limitations of the current study warrant consideration. There was a potential issue of recall bias. Some participants reported AVH experienced years prior. However, recall bias is an unavoidable issue for research into AVH to progress. Attempts were made to control for this by excluding persons who were unable to clearly recount details of their AVH experiences. Though it may have limited bearing on self‐report accuracy, participants who reported historic voices were typically those who shared that they could remember the experience with heightened clarity. There is also a possible influence of mood at time of interview on the reliability of self‐report, given some participants met thresholds for probable mania, hypomania or depression (Teasdale & Russell, 1983). Several issues relating to sample representativeness must also be recognised, as they may limit the generalisability of findings to BD more widely. One of the inclusion criterion was the presence of AVH at least once a day over 3 distinct episodes meaning the participants in the study had to have multiple episodes of hallucinations. This was selected as an inclusion criteria to aid recall; however, it limits the generalisability of the findings given the diversity in AVH experiences in BD. The study included mixed diagnostic subtypes of BD, although this is somewhat representative of the heterogeneity of the disorder. A cross‐sectional approach was adopted, posing unique problems in fully characterising AVH. Even validated instruments for AVH assessment had limitations as applied to the current sample. For instance, the MUPS lacked a structured longitudinal component or reliable way to assess highly changeable AVH experiences and congruence of voice experiences with mood was self‐reported. Inclusion of two international sites offered benefits as well as drawbacks. Participant numbers were bolstered, and wider recruitment avenues likely increased representativeness, although differing researchers and (some) measures possibly introduced a separate source of bias.

### Future research

Current findings have broader implications for research into AVH and their treatment in BD. There was a considerable range in estimated age of AVH onset. A lack of such information has been previously identified as a focus for future research (Toh et al., 2015). Replication in a larger sample to ascertain how AVH phenomenology may differ based on age at index experience is also worthwhile. A proportion of the current sample noted that the tone and content of their voices had changed over time. The concept of evolving AVH, or ‘dynamic developmental progression’, is considered poorly understood (Jones, 2010). Current findings would support research into AVH progression, principally in BD, where marked variations in voice frequency, form and content relative to mood states exist. Participants reported positive and negative emotions following AVH experiences as well, but data captured do not allow for more specific analysis of affective impact. Longitudinal research into AVH in BD may thus contribute to the examination of putative dynamic interactions between mood and AVH experiences, as well as other psychotic features including delusions (Copolov et al., 2004).

Currently recommended cognitive behavioural treatments for voices address a range of cognitive processes known to be influential in AVH initiation and maintenance, for example involving coping, misattribution, perceived power/control and trauma memories (McCarthy‐Jones, 2015). However, it is not clear which aspects or features of AVH these processes underpin (Smailes et al., 2015). Better taxonomies of AVH might therefore facilitate clearer links between specific AVH features with cognitive and neurobiological mechanisms, leading to more tailored interventions (Thomas, 2015). Recent attempts to develop more phenomenologically defined subcategories of AVH should not overlook the diversity of voices that may be significant in BD (McCarthy‐Jones et al., 2014). It is possible that these less well‐described AVH subtypes may form part of the dynamic of mood shifts in a clinically significant way, perhaps escalating mania behaviours or depressive withdrawal by concurring with and inciting an individual's thoughts or actions.

## CONCLUSION

The clinical heterogeneity of BD and associated methodological challenges that accompany the study of symptoms occurring within acute mood states means that AVH phenomenology and its impact on persons with BD have been somewhat neglected in research. Yet this provisional study has indicated a possible dynamic interaction between AVH form and content with relative mood states, as well as subtle differences in individual experiences that may be clinically relevant and merit further scrutiny.

## AUTHOR CONTRIBUTIONS

Author Smith performed statistical analysis and wrote up the first draft of the manuscript. Author Toh assisted with statistical analysis as well as write‐up and populated the tables. All authors provided intellectual and editorial input and have approved the final article.

## CONFLICT OF INTEREST STATEMENT

The authors whose names are listed below certify that they have NO affiliations with or involvement in any organisation or entity with any financial interest (such as honoraria, educational grants, participation in speakers' bureaus, membership, employment, consultancies, stock ownership or other equity interest, and expert testimony or patent licensing arrangements), or nonfinancial interest (such as personal or professional relationships, affiliations, knowledge or beliefs) in the subject matter or materials discussed in this manuscript.

## Supporting information


**Table S1.** Proportions of participants endorsing phenomenological facets of auditory hallucinations based on select MUPS items (*n* = 21)

## Data Availability

The dataset is available on request by qualified researchers‐scientists. Requests require a concept proposal describing the purpose of data access, appropriate ethical approval, and provision for data security. All data analysis scripts and results files are available for review.
