# Supplementary material for: The phenomenology of auditory verbal hallucinations in bipolar disorder
Source: Psychol Psychother. 2023 Feb 7;96(2):399–409. doi: 10.1111/papt.12446 (PMC10952547; doi:10.1111/papt.12446)
Supplement: Supplementary file 1 — Table S1. Proportions of participants endorsing phenomenological facets of auditory hallucinations based on select MUPS items (n = 21) [file PAPT-96-399-s001.docx]

Table A

*Proportions of participants endorsing phenomenological facets of auditory hallucinations based on select MUPS items (n=21)*

| **Age of voice onset (Item 2)**  **Most recent occurrence (Item 3)**^ꝉ^  **Frequency (Item 4)**  **Location (Item 8)**  **Number of voices (Item 9)**^ꝉ^  **Typical volume (Item 11a)**  **Loudest volume (Item 11b)**  **Tone (Item 12a)^ǂ^**  **Tone change over time (Item 12b)**  **Clarity (Item 13a)**^ꝉ^  **Incompleteness (Item 13b)**  **Form of address (Item 16)**  **Voices conversing (Item 19)**  **Running commentary (Item 21)**^ꝉ^  **Content (Item 22a)^ǂ^**  **Content change over time (Item 22b)**^ꝉ^  **Commands (Item 25a)**  **Resistance (Item 25b)**^ꝉ^  **Reality (Item 27)**  **Mood congruence (Item 37)** | 19.0% *<10 years old*; 9.5% *10-14 years old*; 14.3% *15-19 years old*; 23.8% *20-24 years old*; 14.3% *25-29 years old*; 19.0% *>30 years old*  28.6% *Currently*; 9.5% *Past month*; 4.8% *Past three months*; 4.8% *Past six months*; 4.8% *Past 12 months*; 28.6% *>12 months ago*  28.6% *Rarely (0-5 times a week)*; 4.8% *Occasionally (6-10 times a week/once a day)*; 19.0% *Often (11-20 times a week)*; 47.6% *Constantly with you*  33.3% *Inside*; 33.3% *Outside*; 33.3% *Both*  47.6%% *One voice*; 9.5% *Two voices*; 9.5% *More than two voices*; 4.8% *A crowd of voices*; 9.5% *Varies*  9.5% *Whisper*; 57.1% *Normal/conversational*; 23.8% *Loud*; 9.5% *Varies*  4.8% *Too faint to be* audible; 38.1% *Normal/conversational*; 28.6% *Loud*; 28.6% *Yelling/screaming*  47.6% *Harsh*; 33.3% *Angry*; 4.8% *Crackly*; 28.6% *Gentle*; 52.4% *Authoritative*; 33.3% *Bossy;* 42.6% *Malicious/nasty*; 4.8% *Muted;* 9.5% *Muffled;* 4.8% *Indistinct/fuzzy;* 52.4% *Sharp*; 14.3% *Loving*; 28.6% *Kind*; 28.6% *Friendly*; 14.3% *Quiet*; 33.3% *Menacing*  23.8% *Yes*; 71.4% *No;* 4.8% *Unsure*  47.6% *Very sharp/unusually clear*; 42.9% *Clear*; 4.8% *Varies*  38.1% *Yes*; 61.9% *No*  4.8% *First-person*; 52.4% *Second-person*; 19.0% *Implied second-person*; 4.8% *Third-person*; 14.3% *Varies*; 4.8% *Other*  38.1% *Never*; 19.0% *Rarely*; 19.0% *Sometimes*; 23.8% *Often*  38.1% *Never*; 9.5% *Rarely*; 19.0% *Sometimes*; 28.6% *Often*  38.1% *Persecutory*; 33.3% *Abusive/insulting*; 9.5% *Obscene*; 9.5% *Helpful*; 19.0% *Guiding*; 23.8% *Affirming*; 52.4% *Intrusive*; 9.5% *Changeable*; 47.6% *Derogatory*; 38.1% *Accusatory*; 19.0% *Threatening*; 14.3% *Inspiring*; 57.1% *Critical*  38.1% *Yes*; 57.1% *No;* 4.8% *Unsure*  38.1% *Never*; 9.5% *Rarely*; 23.8% *Sometimes*; 28.6% *Often*  9.5% *Never*; 14.3% *Rarely*; 23.8% *Sometimes*; 14.3% *Often*  4.8% *Dream-like*; 4.8% *Somewhat real*; 85.7% *Very real*; 4.8% *Depends*  42.9% *Never*; 4.8% *Rarely*; 23.8% *Sometimes*; 9.5% *Often*; 19.0% *Unsure/not necessarily* |
| --- | --- |

*Note.* MUPS=Mental Health Research Institute Unusual Perceptions Schedule. ^ꝉ^Where the cumulative percentages add up to less than 100%, this reflects missing or incomplete responses; ^ǂ^where the cumulative percentages add up to more than 100%, this reflects the endorsement of multiple response options.
